# Isoleucine Regulates the Synthesis of Pancreatic Enzymes via the Activation of mRNA Expression and Phosphorylation in the Mammalian Target of Rapamycin Signalling Pathways in Pancreatic Tissues

**DOI:** 10.1155/2019/6302950

**Published:** 2019-06-18

**Authors:** Yangchun Cao, Kai Liu, Shimin Liu, Long Guo, Chuanjiang Cai, Junhu Yao

**Affiliations:** ^1^College of Animal Science and Technology, Northwest A&F University, Yangling 712100, China; ^2^UWA School of Agriculture and Environment, the University of Western Australia, Crawley, WA 6009, Australia

## Abstract

This study aimed to investigate the effects of isoleucine (Ile) on the synthesis and secretion of digestive enzymes and cellular signalling in the pancreatic tissue of dairy goats. The pancreatic tissues were incubated in buffer containing 0, 0.40, 0.80, and 1.60 mM Ile. High levels of Ile significantly increased the buffer release and total concentration of *ɑ*-amylase in the tissues (P < 0.001). The total trypsin and chymotrypsin concentrations in each of the Ile groups were significantly higher than those in the control group (P < 0.05); however, lipase was not affected. High levels of Ile significantly increased *ɑ*-amylase mRNA expression (P < 0.001) but had no effect on the mRNA expression of trypsin, chymotrypsin, or lipase. Ile did not affect S6K1 phosphorylation levels. High levels of Ile significantly increased the expression of the *γ* isoform of 4EBP1 (P < 0.001), which indicated that the phosphorylation of 4EBP1 was significantly increased. The phosphorylation level of eEF2 gradually decreased with the addition of Ile (P < 0.001). These results suggested that high doses of Ile can regulate the excretion of enzymes, especially *ɑ*-amylase, in the pancreatic tissues of dairy goats by modulating mTOR signalling, and this regulation is independent of the mTOR-S6K1 pathway.

## 1. Introduction

The specificity of ruminal digestion is different from that of monogastric animals. Studies on the digestive functions of ruminants have thus mostly concentrated on the rumen, whereas relatively few studies exist on the metabolism of nutrients that bypass the rumen. The digestion of rumen bypass nutrients depends on the digestive enzymes secreted by the intestine and other organs, including amylase, oligosaccharides, polysaccharide-hydrolysing enzymes, trypsin, chymotrypsin, and lipase, among others, of which pancreatic exocrine digestive enzymes (*ɑ*-amylase, trypsin, and lipase) predominate. Recent studies have shown that inadequate secretion of pancreatic *ɑ*-amylase might limit intestinal starch utilization in ruminants [1, 2]. The energy utilization efficiency of starch degraded in the small intestine is 42% higher compared with its fermentation products in the rumen, which can help to avoid rumen acidosis [3]. Therefore, improving the exocrine function of the pancreas is conducive to improving the digestion and utilization of rumen bypass nutrients. Proteins and amino acids play important roles in animal energy balance, protein synthesis, function, and substance transformation, and functional amino acids (leucine, isoleucine, phenylalanine, etc.) can be used as signalling molecules to control metabolic function in animals [4, 5].

An increasing number of scholars have begun to focus on the study of branched-chain amino acids. Branched-chain amino acids are a group of neutral, aliphatic amino acids with a branched carbon chain structure and include leucine, isoleucine (Ile) and valine [6]. Leucine is a ketogenic amino acid, Ile is both glucogenic and ketogenic, and proline is a glucogenic amino acid [6]. Animals cannot synthesize branched-chain amino acids and the intake of these amino acids occurs only through food. Branched-chain amino acids mainly have physiological functions including repairing muscles, promoting gluconeogenesis, oxidizing and supplying energy for the body, enhancing immunity, and promoting hormone synthesis and secretion [7, 8]. Branched-chain amino acids are functional amino acids. Our research group has devoted many years to the study of functional amino acids and pancreatic exocrine function in ruminants, especially leucine [9–11]; however, Ile, a similar branched-chain and functional amino acid, has been rarely investigated. Like leucine, Ile not only is a substrate for protein synthesis but also plays an important role in intracellular protein synthesis signalling pathways [7]. A study in monogastric animals showed that Ile increased milk protein and milk fat production rates in sows [8]. Previous studies in fish showed that Ile increased the growth rate, feed intake, protein synthesis, and protein deposition in juvenile Jian carps and that the activities of trypsin, chymotrypsin, lipase, and amylase were improved [12]. Ile also has an important regulatory role in protein synthesis. Few studies on Ile in ruminants exist and even fewer studies regarding the effects of Ile on pancreatic functions have been done.

Mammalian target of rapamycin (mTOR) is a highly conserved protein factor that can regulate the growth and metabolism of animals through a mechanism involving the regulation of protein synthesis that is relatively clear [13, 14]. mTOR plays a very important role in the initial stage of translation. Amino acids can regulate protein synthesis by affecting the phosphorylation levels of upstream and downstream target protein molecules in the mTOR pathway [15]. The best-characterized downstream effectors of mTOR include two signaling pathways that act in parallel to control mRNA translation: the 70-kDa ribosomal protein S6 kinase 1 (S6K1) pathway and the eukaryotic translation initiation factor 4E binding protein 1 (4EBP1) pathway [16]. mTOR activated by amino acids, in turn, catalyses the phosphorylation of ribosomal protein S6K1 and 4EBP1 [17]. Another potentially important signaling protein in the control of translation is eukaryotic elongation factor 2 (eEF2), which is indirectly regulated by mTOR through S6K1 [18]. In mammalian epithelial cell culture assays [19, 20], the level of mTOR phosphorylation was approximately 100% higher in the 3.5 mM essential amino acid group compared to the nonessential amino acid group (P < 0.001). With the removal of Ile alone, the level of mTOR phosphorylation was decreased by 47% (P < 0.05). Under energy or amino acid depletion conditions, intracellular mTORC1 signalling is strongly inhibited, while the resupplementation of amino acids to starved cells can significantly stimulate mTORC1 activity [8]. However, the mechanisms by which amino acids act as mTORC1 signalling factors remain unclear.

The aim of this study was to investigate the molecular mechanisms and signalling pathways associated with Ile and the regulation of pancreatic exocrine function.

## 2. Materials and Methods

All procedures used in this study complied with an animal care protocol that was approved by the Northwest A&F University Animal Care and Use Committee.

### 2.1. Pancreatic Tissue Preparation

Pancreatic tissue was obtained from three different, 1-year-old, healthy Guanzhong dairy goats. A total mixed ration (Table 1) composed of alfalfa hay (17.5% of dry matter (DM)), corn silage (27.5% of DM), and concentrate (55.0% of DM) was prepared for the goats. Goats were fed twice daily at 0700 and 1900 h on an ad libitum basis (allowing for 5-10% orts) with free access to fresh water. Three goats were slaughtered over 3 days, one goat per day, to provide fresh pancreatic tissues for culturing.

Approximately 10 g of pancreas tissue was quickly excised after animal slaughtering, placed in cold normal saline (0.9% NaCl), and immediately sent to the laboratory. The tissue was transferred to KRB buffer (25 mM HEPES, pH 7.4, 118 mM NaCl, 4.7 mM KCl, 1.2 mM MgSO_4_, 2.5 mM CaCl_2_, and 1.2 mM KH_2_PO_4_) [21] and cut into pieces 2 × 2 mm in size. After moisture removal with absorbent papers, tissue pieces were weighed and then transferred to a culture bottle containing 3 mL of KRB solution. The culture bottle was placed in a cell incubator (95% O_2_ /5% CO_2_) with the bottle cap left open for incubation. After incubation, the culture bottle was placed on ice and the buffer was collected and stored frozen. The buffer was used to determine the amount of enzymes released from the pancreatic tissue. The pancreatic tissue was frozen and then used to determine the enzyme activities (*ɑ*-amylase, trypsin, and lipase), as well as the mRNA abundance and activity and the degree of phosphorylation of the mTOR signalling pathway factors (4EBP1, S6K1, and eEF2).

### 2.2. Treatments and Experimental Design

The Ile solutions containing Ile at 0 mg/mL, 2.62 mg/mL, 5.24 mg/mL, and 10.48 mg/mL were prepared in KRB buffer and were added to cell culture medium to achieve final Ile concentrations of 0 mM, 0.40 mM, 0.80 mM, and 1.60 mM, respectively. The incubation time was 180 min and three replicates were included for each treatment. After incubation for 180 min, tissues were harvested by scraping in the presence of ice-cold lysis buffer containing 1% (v:v) of protease and phosphatase inhibitors cocktail (Roche, China). The culture medium was also collected for further analysis of enzyme activities. In total, each Ile treatment included three replicates from three goats (n = 3).

### 2.3. Sample Analysis

#### 2.3.1. Chemical Composition of Diet

Feed samples were collected, dried at 55°C for 72 h, and then ground through a 1 mm screen. These samples were analysed using the procedures described by AOAC (1999) for dry matter (DM, ID 930.5) and crude protein (CP, N×6.25; ID 984.13) [22]. The neutral detergent fibre (NDF) and acid detergent fibre (ADF) contents were determined according to the method of Van Soest et al. [23] using sodium sulphite and a heat-stable *α*-amylase (Ankom A200I fibre analyser, Ankom Technology, Macedon, NY). The starch content in the bag was determined using an enzymatic method (*α*-amylase and amyloglucosidase) with a commercial starch analysis kit (Megazyme, Megazyme International Ireland Ltd., Bray, Ireland).

#### 2.3.2. *In Vitro* Enzyme Release

To measure the activity of the digestive enzymes in the tissue segments, the homogenate supernatant was collected after homogenizing. The activity of *α*-amylase, trypsin, and lipase in the supernatant and culture medium were determined using commercial kits (Nanjing Jiancheng Bioengineering Institute, China). One unit of enzyme activity was defined as 1 *μ*mol of the product released per minute at 39°C.

#### 2.3.3. Quantification of Amylase, Trypsin, and Lipase mRNA Levels

RNA extraction was performed according to the method described by Wathes et al. [24]. Frozen pancreatic tissue samples were homogenized with 1 mL of Trizol (Invitrogen, USA) and the supernatants were collected by centrifugation. RNA was precipitated by adding chloroform, followed by isopropanol. The RNA pellet was rinsed with 75% ethanol and dissolved in RNAse-free water. Total RNA was measured using 260 nm spectrometry.

Reverse transcription was performed using the RNA PCR Kit 3.0 (TAKaRa Biotechnology (Dalian) Co. Ltd., China). Solutions included 2 *μ*L of MgCl_2_, 3.75 *μ*L of RNAse-free water, 1 *μ*L of 10× RT buffer, 0.25 *μ*L of RNAse inhibitor, 1 *μ*L of dNTP mix, 0.5 *μ*L of random primers, 0.5 *μ*L of AMV reverse transcriptase, and 1 *μ*L of the RNA sample. The reaction conditions were as follows: 42°C for 30 min, 95°C for 5 min, 5°C for 5 min, and storage at −20°C.

For quantitative real-time PCR, 25 *μ*L of the reaction system containing SYBR Green (TAKaRa Biotechnology (Dalian) Co. Ltd., China) was used. The reaction solution contained 12.5 *μ*L of 2× SYBR premix Ex Taq II, 2 *μ*L of sample cDNA, 8.5 *μ*L of dH_2_O, 1 *μ*L of 10 *μ*mol/L forward primer, and 1 *μ*L of 10 *μ*mol/L reverse primer. The reaction conditions were as follows: predenaturation at 95°C for 30 s, 40 cycles of denaturation at 95°C for 5 s, annealing at 63°C for 30 s (different temperatures for different primers), and final extension at 72°C for 10 min. Primers for *ɑ*-amylase, trypsin, chymotrypsin, lipase, and *β*-actin are listed in Table 2 [4]. mRNA expression was determined using the 2-^ΔΔ^CT method [25] with *β*-actin used as the housekeeping gene [26].

#### 2.3.4. Protein Immunoblot Analysis

Western blot analysis was performed according to the methods described by Crozeir et al. [27]. The frozen tissue samples were ground to powders with a mortar (under a liquid nitrogen environment) and then homogenized using 2 mL of cold lysis buffer (pH = 7.4). The lysis buffer contained the following: 50 mM TrisHCl, 25 mM sodium fluoride, 5 mM ethylenediaminetetraacetic acid, 50 mM *β* -glycerophosphate, 10 mM sodium pyrophosphate, 0.2 mM sodium vanadate, 1 mM benzylsulfonyl fluoride, 0.2% polyethylene glycol octyl phenyl ether (v/v), 1 mM dithiothreitol, 10 *μ*g/mL aprotinin, 5%  *β*-mercaptoethanol (v/v), and 10 *μ*g/mL leupeptin. Upon homogenization, the solution was immediately centrifuged (20000 × g, 4°C, 15 min) to collect the supernatant, which was then boiled for 15 min and centrifuged a second time (10000 × g, 4°C, 30 min). The new supernatant was collected for use in immunoblot analyses to determine 4EBP1, S6K1, and eEF2 expression. Prior to western blot analysis, the sample was boiled for 5 min and then cooled on ice. The western blot procedure was as follows: first, protein separation was conducted based on the protein molecular weight using sodium dodecyl sulphate-polyacrylamide gel electrophoresis (SDS-PAGE) with a polyacrylamide gel concentration of 15%. Then, the protein was transferred to a nitrocellulose membrane and the band of interest was incubated with the corresponding primary antibody for 24 h at 4°C. Next, the membrane was rinsed three times with phosphate buffered saline (PBS) and incubated with secondary antibody for 1 h at room temperature. Finally, the ChemiDOC XRS+ imaging system (Bio-Rad, Germany) was used to develop the protein of interest through enhanced chemiluminescence reactions. The primary antibodies used were rabbit anti-goat polyclonal serum antibodies (4EBP1, Calbiochem, Germany; S6K1 and eEF2, Abcam, UK) and the secondary antibodies were goat anti-rabbit horseradish peroxidase conjugates (Beijing Synthetic Technology Co., Ltd., China).

### 2.4. Calculations and Statistical Analysis

Enzyme activities, mRNA expression, and 4EBP1, S6K1 and eEF2 phosphorylation were analysed using the GLM procedure for the one-way analysis of variance (ANOVA) model with SAS software. Protein expression content was calculated as the ratio of the band intensity of *β*-actin. Differences of P < 0.05 were considered significant and data are presented as the means ± standard errors of the means (SEM).

## 3. Results

As shown in Table 3, high levels of Ile (0.8 mM and 1.6 mM) significantly increased the tissue concentration of *ɑ*-amylase (P < 0.001), the buffer release concentration (P < 0.001), and the total concentration (P < 0.001). The total concentrations of trypsin and chymotrypsin in each of the Ile groups were significantly higher than those in the control group (P < 0.05), while lipase was not affected by Ile (P > 0.05).

**Figure 1:**
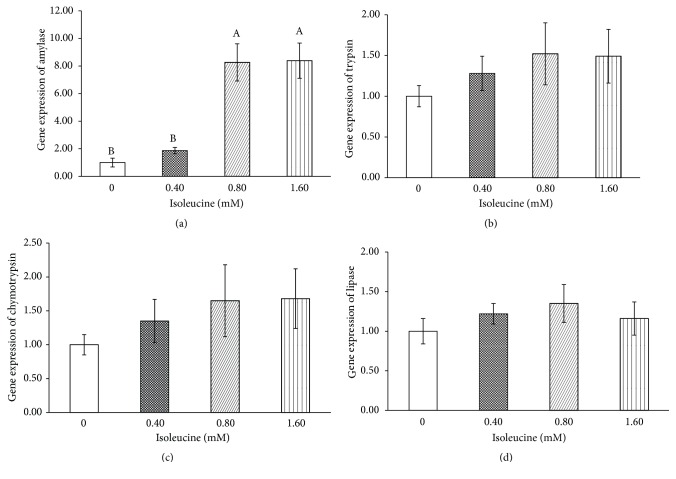
The effects of isoleucine on pancreatic amylase (a), trypsin (b), chymotrypsin (c), and lipase (d) mRNA levels* in vitro*. The values are the means and pooled standard errors of the means (SEM). The enzyme mRNA levels were normalized to *β*-actin and the values were compared to the control group, which was set to 1.00. Different letters represent significantly different values (*P* < 0.05, n = 3).

As shown in Figure 1, high levels of Ile (0.8 mM and 1.6 mM) significantly increased *ɑ*-amylase mRNA expression (P < 0.001), but had no effect on the mRNA expression of trypsin, chymotrypsin, or lipase (P > 0.05).

**Figure 2:**
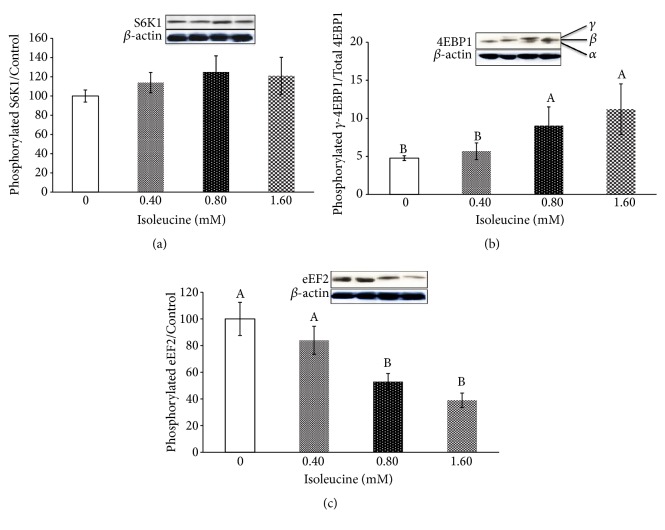
The effects of isoleucine on the ratio of phosphorylated to total mTOR signalling pathway factors in pancreas tissue. Panel (a) represents S6K1; Panel (b) represents 4EBP1 (*ɑ*, *β*, and *γ*-forms are denoted); Panel (c) represents eEF2. The data are the means and pooled standard errors of the means (SEM). Different letters represent significantly different values (*P* < 0.05, n = 3).

As shown in Figure 2, Ile did not affect the level of S6K1 phosphorylation (P > 0.05). Detection of the 4EBP1 isoforms (*ɑ*, *β*, and *γ*) indicated that high levels of Ile (0.8 mM and 1.6 mM) significantly increased the expression of the *γ* isoform of 4EBP1 (P < 0.001), which means that the phosphorylation of 4EBP1 was significantly increased. The level of eEF2 phosphorylation gradually decreased with the addition of Ile and was most pronounced in the 0.8 mM and 1.6 mM groups (P < 0.001).

## 4. Discussion

In this study, Ile significantly increased the total *ɑ*-amylase concentration in pancreatic tissues. Compared to the control group, the total *ɑ*-amylase concentrations in the 0.8 mM and 1.6 mM groups were increased by 346.3% and 289.4%, respectively. A study on juvenile Jian carp noted that Ile significantly increased the trypsin, chymotrypsin, and *ɑ*-amylase activities in the hepatopancreas and intestines [12], which was similar to the results of a study in mice by Lyman and Wilcox [28]. A previous study conducted by our research team [29] also showed that short-term duodenal infusion of Ile could increase the *ɑ*-amylase concentration (U/mg) and the secretion rate (U/h) by up to 84.6% and 78.6%, respectively, and long-term infusion could increase the same parameters by 122.7% and 133.8%, respectively. The reason why Ile has different degrees of stimulatory effects on the synthesis and secretion of *ɑ*-amylase may be because when nutrient infusion is performed in animals, pancreatic enzyme secretion is affected by various factors, including nutrients, hormones, nerves, and gastrointestinal motility, as well as the interactions and antagonistic effects among these factors [2]. By contrast, with* in vitro* incubation of pancreatic tissues, other impacting factors in* in vivo* studies can be ruled out and the effects of nutrients on pancreatic enzyme secretion can be studied in isolation, producing a more direct and obvious effect. Low-dose Ile has no effect on pancreatic enzyme secretion. The reason for this remains to be further investigated as few studies regarding the effects of Ile on pancreatic exocrine functions in ruminants exist and no current studies regarding the effects of Ile on enzyme secretion in* in vitro* pancreatic tissues or acinar cells in ruminants are known. The effects of Ile on pancreatic enzyme mRNA expression may, to a certain extent, explain that low-dose Ile had no effect on *ɑ*-amylase secretion in the pancreas. High levels of Ile (0.8 mM and 1.6 mM) promoted *ɑ*-amylase mRNA expression and the effect was extremely significant.

Amino acids not only serve as substrates for protein synthesis but also regulate protein synthesis by modulating translation initiation and translation rates [30]. Previous studies have shown that Ile can increase mTOR phosphorylation and the synthesis rate of casein fragments in mammary gland tissues [19, 20]. Arriola Apelo et al. also showed that Ile can stimulate the mTOR signalling pathway in mammary gland tissues [31]. In this study, Ile significantly increased the level of 4EBP1 phosphorylation. 4EBP1 acts as a downstream signalling factor of mTOR and is directly regulated by mTOR [14]. Once being phosphorylated by mTOR, 4EBP1 releases eukaryotic initiation factor 4E (eIF4E) from the inactive eIF4E·4EBP1 complex to form the active eIF4G·eIF4E complex that binds to mRNA and initiates translation [32]. Therefore, Ile can regulate protein synthesis by modulating the mTOR signalling pathway. However, since Ile does not affect the level of S6K1 phosphorylation, the results of this study indicate that the regulation of protein synthesis by Ile does not depend on the mTOR-S6K1 pathway.

eEF2 plays a crucial role during the elongation stage of protein synthesis, in which the ribosome moves by the equivalent of one codon relative to the mRNA, and the peptidyl-tRNA migrates from the ribosomal A site into the P site, following formation of the new peptide bond [16], while phosphorylated eEF2 is inactive during protein translation [18]. Studies have shown that amino acid deficiency increases the degree of eEF2 phosphorylation [33]. By studying the effects of different amino acids on the protein synthesis pathway in mammary gland tissues, Arriola Apelo et al. [31] showed that Ile can linearly reduce the level of eEF2 phosphorylation and thus increase its activity. A study by Proud et al. showed that eEF2 can be regulated by mTOR [34]. In this study, as the level of Ile increased, the degree of eEF2 phosphorylation gradually decreased, with a particularly significant effect under high-doses of Ile, which was consistent with the above findings.

## 5. Conclusions

High doses of Ile can regulate the excretion of enzymes, especially *ɑ*-amylase, in dairy goat pancreatic tissue by modulating mTOR signalling and this regulation is independent of the mTOR-S6K1 pathway.

## Figures and Tables

**Table 1:** Ingredients and chemical composition of the experimental diet.

Item	% (DM)
Ingredient	
Alfalfa hay	17.50
Corn silage	27.50
Corn	40.00
Soybean meal	13.00
Calcium phosphate	0.25
Limestone	0.75
NaCl	0.50
Vitamin-mineral premix^1^	0.50
Nutrient composition	
DM	50.58
ADF	18.88
NDF	34.38
CP	16.40
Starch	27.66

^1^Vitamin-mineral premix (per kg): 600 mg of Mn, 950 mg of Zn, 430 mg of Fe, 650 mg of Cu, 30 mg of Se, 45 mg of I, 20 mg of Co, 450 mg of nicotinic acid, 800 mg of vitamin E, 45,000 IU of vitamin D, and 120,000 IU of vitamin A.

**Table 2:** Real-time PCR primers.

Gene name	Reference sequence	Primer sequence (5′-3′)	Product size (bp)	Annealing temperature (°C)
Amylase	NM_001035016	F: GAAATGGCCGTGTGACAGAATTTA	142	64.3
		R: ACAAAGACAAGTGCCCTGTCAGAA		
Trypsin	NM_001113727	F: TGTCTGCGGCTCACTGCTAC	119	62.7
		R: GCTGGGATGGACGATACTCTTG		
Chymotrypsin	NM_001098965.1	F: ATGTTGGGCATCACGGTCTT	172	60.0
		R: TGTGCCTCCACGTGTTATCC		
Lipase	NM_001205820	F: GTGGAAGCAAATGATGGACAAG	81	61.8
		R: TGGGTTGAGGGTGAGCAGA		
*β*-Actin	AF481159	F: ACCACTGGCATTGTCATGGACTCT	152	60.0
		R: TCCTTGATGTCACGGACGATTTCC		

**Table 3:** The effects of isoleucine on the activities of enzymes *in vitro*.

Item	Level of isoleucine (mM^1^)				SEM^2^	P-value
	0	0.40	0.80	1.60		
Tissue concentration (U/g)						
*ɑ*-amylase	814^b^	923^b^	4207^a^	3631^a^	244.7	< 0.001
Trypsin	4.45	5.13	4.87	4.91	0.445	0.148
Chymotrypsin	3.51	4.12	4.32	4.28	0.629	0.093
Lipase	587	634	608	616	88.6	0.347
Release (U/g tissue)						
*ɑ*-amylase	325^b^	308^b^	876^a^	804^a^	48.3	< 0.001
Trypsin	1.92	2.14	2.06	2.26	0.318	0.271
Chymotrypsin	1.22	1.56	1.48	1.49	0.475	0.248
Lipase	189	195	213	204	49.7	0.418
Total activity (U/g tissue)						
*ɑ*-amylase	1139^b^	1231^b^	5083^a^	4435^a^	138.1	< 0.001
Trypsin	6.37^b^	7.27^a^	6.93^a^	7.17^a^	0.582	0.014
Chymotrypsin	4.73^b^	5.68^a^	5.80^a^	5.77^a^	0.536	0.028
Lipase	776	819	821	820	72.3	0.303

^1^One-way ANOVA. Differences were considered significant at P < 0.05.

^2^Pooled standard error of the means, n=3.

## Data Availability

The data used to support the findings of this study are available from the corresponding author upon request.
